# Apoptosome activation, an important molecular instigator in 6-mercaptopurine induced Leydig cell death

**DOI:** 10.1038/srep16488

**Published:** 2015-11-18

**Authors:** Jessica A. Morgan, John Lynch, John C. Panetta, Yao Wang, Sharon Frase, Ju Bao, Jie Zheng, Joseph T. Opferman, Laura Janke, Daniel M. Green, Wassim Chemaitilly, John D. Schuetz

**Affiliations:** 1 Departments of Pharmaceutical Sciences, St. Jude Children’s Research Hospital, 262 Danny Thomas Place, Memphis, TN 38105; 2 Cellular Imaging Shared Resource, St. Jude Children’s Research Hospital, 262 Danny Thomas Place, Memphis, TN 38105; 3 Structural Biology, St. Jude Children’s Research Hospital, 262 Danny Thomas Place, Memphis, TN 38105; 4 Cell and Molecular Biology, St. Jude Children’s Research Hospital, 262 Danny Thomas Place, Memphis, TN 38105; 5 Pathology, St. Jude Children’s Research Hospital, 262 Danny Thomas Place, Memphis, TN 38105; 6 Epidemiology & Cancer Control, St. Jude Children’s Research Hospital, 262 Danny Thomas Place, Memphis, TN 38105; 7 Endocrinology, St. Jude Children’s Research Hospital, 262 Danny Thomas Place, Memphis, TN 38105.

## Abstract

Leydig cells are crucial to the production of testosterone in males. It is unknown if the cancer chemotherapeutic drug, 6-mercaptopurine (6 MP), produces Leydig cell failure among adult survivors of childhood acute lymphoblastic leukemia. Moreover, it is not known whether Leydig cell failure is due to either a loss of cells or an impairment in their function. Herein, we show, in a subset of childhood cancer survivors, that Leydig cell failure is related to the dose of 6 MP. This was extended, in a murine model, to demonstrate that 6 MP exposure induced caspase 3 activation, and the loss of Leydig cells was independent of Bak and Bax activation. The death of these non-proliferating cells was triggered by 6 MP metabolism, requiring formation of both cytosolic reactive oxygen species and thiopurine nucleotide triphosphates. The thiopurine nucleotide triphosphates (with physiological amounts of dATP) uniquely activated the apoptosome. An ABC transporter (Abcc4/Mrp4) reduced the amount of thiopurines, thereby providing protection for Leydig cells. The studies reported here demonstrate that the apoptosome is uniquely activated by thiopurine nucleotides and suggest that 6 MP induced Leydig cell death is likely a cause of Leydig cell failure in some survivors of childhood cancer.

Leydig cells in the testes are the primary source of testosterone production in males^1^ and have a crucial endocrine function. Leydig cell failure (LCF), characterized by raised levels of luteinizing hormone and reduced systemic testosterone^2^,^3^, reportedly affects 10 to 60% of childhood cancer survivors but has primarily been reported in patients who received either high dose alkylating agent chemotherapy or doses of radiotherapy in excess of 20 Gy^1^,^2^,^3^,^4^,^5^,^6^,^7^. In a report from the St. Jude Lifetime (SJLIFE) cohort, the prevalence of LCF was 11.5% among adults who were cured of childhood cancer by treatment that included alkylating agents or testicular radiotherapy^4^. However, the frequency of LCF might be underestimated because knowledge on the effect of other cancer chemotherapy treatments on Leydig cells is incomplete or unknown. Given the estimated 360,000 childhood cancer survivors^5^ and the potential impact of chemotherapy on the quality of life, it is incumbent that we determine the prevalence and mechanism of Leydig cell dysfunction that occurs among those cancer survivors who have received other chemotherapeutic agents^5^.

We focused on the antimetabolite 6 MP, a mainstay of modern cancer therapy^6^,^7^,^8^ that has dramatically increased acute lymphoblastic leukemia (ALL) survival rates, but it was unclear if 6 MP therapy affected childhood testosterone production in adult ALL survivors^9^,^10^,^11^. The current study was undertaken to determine whether 6 MP impacts Leydig cell survival because Leydig cells are non-proliferating^12^ and it was unknown if 6 MP, a drug also widely used as an immunosuppressant, affected Leydig cell viability.

Typically, thiopurine metabolism in proliferating cells leads to 6-thiodeoxyguanosine (dGS) nucleotide incorporation into DNA, which is considered the primary mechanism of thiopurine cytotoxicity^13^,^14^,^15^. This produces two effects. First, insertion of dGS into DNA^16^ accounts for altered DNA-protein interactions^13^,^17^,^18^. Second, the DNA mismatch repair system (MMR) promotes thiopurine cytotoxicity by initiating a cycle of futile efforts to repair DNA lesions containing thioguanine mismatch pairs^19^,^20^. This is consistent with studies showing the MMR complex binds to S6-dGS:Thymidine mismatches in DNA^21^,^22^. However, currently there is no clear mechanistic explanation for thiopurine mediated killing of non-proliferating cells, such as Leydig cells. The goal of these studies was to first determine if LCF occurred in humans exposed to 6 MP (in individuals that were not exposed to either alkylating agent chemotherapy and/or doses of radiotherapy) and, second, to develop a mechanistic understanding of how Leydig cells are affected by 6 MP.

## Results and Discussion

### Leydig cell failure in patients receiving methotrexate and 6-mercaptopurine

**Figure 1:**
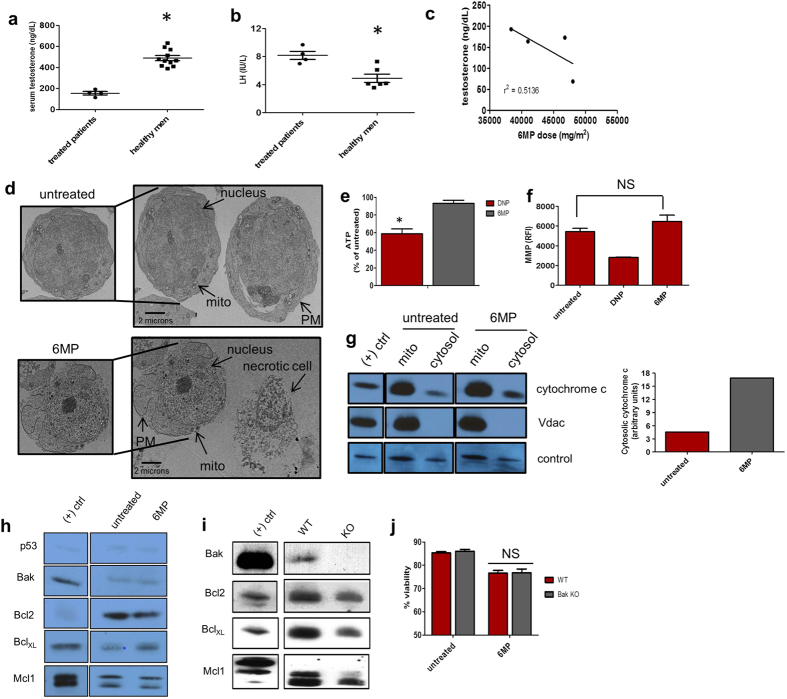
6 MP is toxic to Leydig cells. (**a**) Serum testosterone and (**b**) LH concentrations of SJLIFE treated patients (n = 4) versus published controls (n = 3 independent studies for testosterone^25^,^26^,^27^ and 4 independent studies for LH^29^,^30^,^31^,^32^). (**c**) Serum testosterone concentration vs 6 MP dose administered to SJLife patients. (**d**) TEM of untreated (top) and 6 MP treated (bottom) Leydig cells. Measurements of (**e**) ATP and (**f**) mitochondrial membrane potential in cells exposed to 6 MP. (**g**) Immunoblot analysis of untreated and 6 MP treated Leydig cell fraction probed for cytochrome *c*. Vdac was used as a control for mitochondrial fraction isolation. Bands were normalized to an endogenous band (labeled control, graph shown on right). Immunblot analysis of Bcl_2_ family member proteins in (**h**) wildtype treated with 6 MP and **(i)** Bak-null Leydig cells. (**j**) Leydig cell viability in Bak KO cells treated with 6 MP. NS= not significant All error bars are mean ± SEM. *p ≤ 0.05.

Leydig cells are the primary source of testosterone in males^1^. Of 763 male participants in the childhood cancer survivors program for adult survivors of childhood cancer (St. Jude Lifetime Cohort Study (SJLIFE)^4^,^5^, 71 had evaluations consistent with the diagnosis of Leydig Cell Failure (LCF), defined as high levels of luteinizing hormone (LH) (>7 IU/ml) in the presence of low testosterone (<250 ng/dL). Among those with a history of treatment for acute lymphoblastic leukemia that included the combination of 6 MP and MTX (without exposure to alkylating agent chemotherapy and/or testicular radiotherapy, both of which are known causes of LCF^11^,^23^,^24^), 5.3% had a diagnosis of LCF (see Materials and Methods). These patients’ average testosterone concentration was 155.3 ng/dL + /− 28.1 ng/dL (average age ≈40.6 and range 36.7–51.8), less than one-third of normal (489.4 + /− 22.9 ng/dL)^25^,^26^,^27^ (Fig. 1a) and well below the recently reported value for the 95% confidence interval of male testosterone concentration^28^. Consistent with LCF, the average LH concentration was 8.3 IU/L + /− 1.01 IU/L, which is about 1.7 times higher than the normal level (4.91 + /− 0.55 IU/L)^29^,^30^,^31^,^32^ (Fig. 1b). An inverse relationship between the cumulative dose of 6 MP and testosterone was observed (r^2^ = −0.51) (Fig. 1c). Strikingly, the most profound LCF (69 ng/dL testosterone and 8.7 IU/L LH) was observed in a patient treated with the largest cumulative dose of 6 MP (Supplemental Table 1). Furthermore, this individual was found to have complete azoospermia upon semen analysis. This retrospective analysis suggests Leydig cell failure is possible among some susceptible childhood cancer patients receiving 6 MP. Based on these findings, we hypothesized 6 MP produced LCF by killing Leydig cells.

### 6 MP produces necrotic cell death

We used a murine model to test the hypothesis that 6 MP kills Leydig cells. Transmission electron microscopy (TEM) was used to assess the morphological features of 6 MP-induced cell death. Cultured Leydig cells were treated with either vehicle or 6 MP, followed by TEM analysis. 6 MP treated Leydig cells exhibited cellular swelling and loss of cell plasma membrane integrity (Fig. 1d). Next, intracellular ATP concentrations were determined in cells treated with 6 MP (Fig. 1e). Unlike cells treated with the positive control, the mitochondrial uncoupler, 2-dinitrophenol (DNP), Leydig cells treated with 6 MP did not exhibit reduced ATP concentration (Fig. 1e). Consistent with the lack of change in ATP, the mitochondrial membrane potential (ΔΨm) was also unaffected by 6 MP treatment (Fig. 1f). Unexpectedly, 6 MP treatment strongly increased the amount of cytochrome *c* released into the cytosol (Fig. 1g).

We next investigated the role of the Bcl-2-family in 6 MP-induced death. 6 MP treatment did not reduce the amounts of anti-apoptotic Bcl-2-family members (Bcl-2, Bcl-X_L_ or Mcl-1) in Leydig cells, although Bcl-X_L_ expression was increased by 6 MP treatment (Fig. 1h). Consistent with previous reports, Bax expression was undetectable in Leydig cells (not shown)^33^, whereas Bak was readily detectable (Fig. 1h). To determine if Bak activation contributed to 6 MP-induced Leydig cell death, we utilized the Bak KO mouse^34^. The expression of Bcl-2 family proteins was not increased in Bak WT KO Leydig cells (Fig. 1i). Importantly, the loss of Bak expression did not alter Leydig cell vulnerability to 6 MP induced death (Fig. 1j).

### MRP4 protects Leydig cells against 6 MP

**Figure 2:**
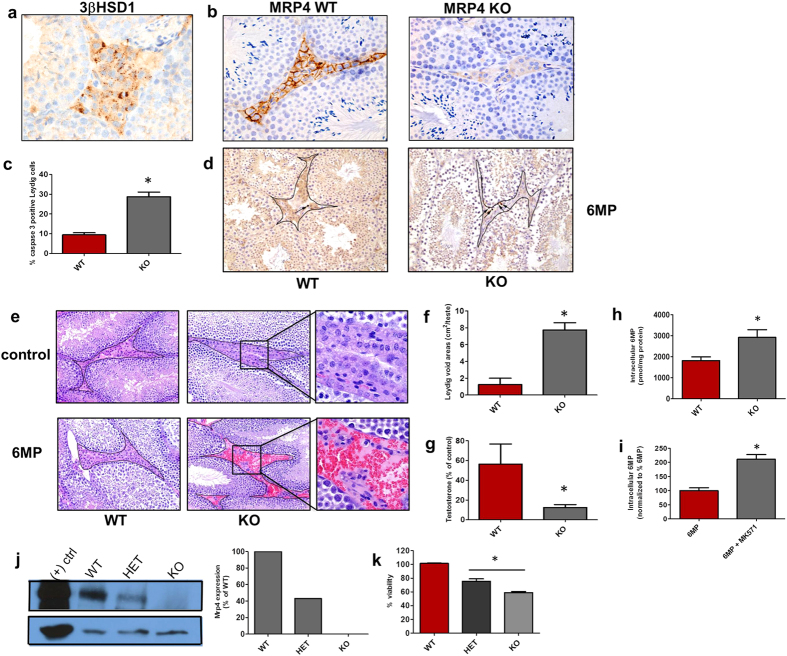
The transporter Mrp4 protects Leydig cells from 6 MP death. Immunohistochemical Leydig cell staining of (**a**) 3βHsd1 and (**b**) Mrp4 in WT (left) and KO (right) mice (magnification ×20). (**c**) Percent of caspase 3 positive Leydig cells of 6 MP treated mice. (**d**) WT (left) and KO (right) 6 MP treated mice showing Leydig cells stained with caspase 3. Dark lines indicate interstitial areas that harbor Leydig cells, between seminiferous tubules. Arrows indicate Leydig cells positively stained for caspase 3 (magnification ×20). **(e)** Control (top) and 6 MP (bottom) treated mice. Dark lines indicate interstitial areas between seminiferous tubules. Enlarged insets show cells in the interstitium. (**f**) Quantitation of void areas represented in (**f**) Serum testosterone concentration of 6 MP treated mice (n = 6 mice). Intracellular accumulation of radiolabelled 6 MP in Leydig cells when comparing WT to (**h**) KO cells or (**i**) WT cells treated with pharmacologic Mrp4 inhibitor, MK571. (**j**) Mrp4 expression in isolated Leydig cells as analyzed by immunoblot (top) and normalized by actin (bottom). The graph on the right shows band quantitation by ImageJ. (**k**) Viability of Mrp4 wildtype (WT), heterozygote (HET) and knockout (KO) Leydig cells treated with 6 MP. All error bars are mean ± SEM. *p ≤ 0.05.

In the testis, Leydig cells are readily identified, immunohistochemically, by the key testosterone biosynthetic enzyme, 3βHSD1 (Fig. 2a). Further, MRP4, a thiopurine nucleotide exporter, is expressed in the plasma membrane of Leydig cells (Fig. 2b). However, it is not known if this amount of MRP4 is sufficient to protect non-replicating Leydig cells from 6 MP induced death.

To investigate the impact of MRP4 on Leydig cells *in vivo*, we used MRP4 knockout (abbreviated KO hereafter) and wild-type (WT) mice that were treated with 6 MP. 6 MP treated KO mice Leydig cells had more extensive cell death vs. WT as shown by the almost 3-fold increase in cleaved caspase 3 immunostaining (Fig. 2c,d). The sensitization of KO Leydig cells to 6 MP was further evident by a 4-fold increase in interstial areas devoid of Leydig cells in the 6 MP treated KO mice (Fig. 2e,f). Consistent with the loss of Leydig cells, the amount of testosterone in the KO testes was reduced over 4-fold (Fig. 2g), similar to our findings for humans treated with 6 MP (see Fig. 1).

Primary cultures of KO and WT Leydig cells were used to determine if MRP4 could reduce 6 MP accumulation. KO Leydig cells accumulated 1.6-fold more 6 MP than WT (Fig. 2h). Consistent with this, the MRP4 inhibitor, MK571^35^, increased intracellular 6 MP accumulation by 2-fold in WT Leydig cells (Fig. 2i). Furthermore, Leydig cell sensitivity to 6 MP was related to the amount of MRP4 as the loss of each MRP4 allele proportionately reduced cell viability (Fig. 2j,k). This increased 6 MP sensitivity was not due to an acquired sensitivity of KO cells to cytotoxins as both KO and WT cells were equally sensitive to etoposide (Supp. Fig. 1).

### 6 MP toxicity associates with ROS generation

**Figure 3:**
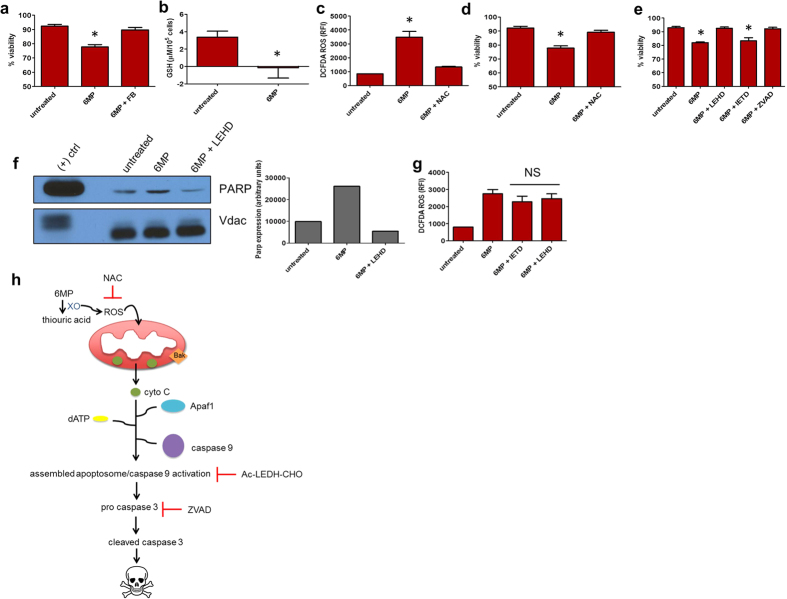
6 MP produces ROS generation and caspase activation (**a**) Leydig cell viability upon exposure to 6 MP with and without the xanthine oxidase inhibitor, febuxostat. (**b**) GSH levels in control and 6 MP treated Leydig cells. (**c**) ROS, and (**d**) viability measurements of 6 MP treated Leydig cells ± ROS scavenger, N-Acetylcysteine (NAC). (**e**) Viability of 6 MP treated Leydig cells ± caspase inhibitors. (**f**) Cleaved PARP protein levels in Leydig cells treated with 6 MP or 6 MP plus a caspase 9 inhibitor. PARP was normalized by Vdac expression (graph on right, quantitated using ImageJ). (**g**) ROS levels in cells treated with either 6 MP plus a caspase 8 (IETD) or caspase 9 inhibitor (LEHD). (**h**) Schematic depicting ROS formation leading to mitochondrial cytochrome *c* release and caspase 9 activation, which can be abrogated by either inhibiting ROS by NAC or caspases by a specific caspase 9 or pan-caspase inhibitor. All error bars are mean ± SEM. *p ≤ 0.05.

In some cell types, 6 MP is metabolized to thiouric acid by cytosolic xanthine oxidase, a process capable of forming reactive oxygen species (ROS), specifically H_2_O_2_^36^. To determine if Leydig cells used xanthine oxidase to metabolize 6 MP, cells were co-treated with febuxostat (labeled “FB”), a specific non-competitive inhibitor of xanthine oxidase^37^, along with 6 MP. Blockade of 6 MP-induced Leydig cell death by FB indicated xanthine oxidase was crucial to initiate Leydig cell death (FB treatment alone produced no change in cell viability) (Fig. 3a). We investigated whether 6 MP affected the level of the ROS scavenger, glutathione (GSH). Consistent with ROS formation, 6 MP treatment profoundly reduced Leydig cell GSH concentration (Fig. 3b) and as expected, the amount of ROS in Leydig cells treated with 6 MP increased 6-fold. Restoring GSH^38^ by treatment with N-acetylcysteine (NAC) both blocked cell death and markedly suppressed ROS in 6 MP treated cells (Fig. 3c). Mitochondrial ROS concentration was only modestly increased by 6 MP, suggesting this change was unrelated to cell death (Supp. Fig. 2). This proposition is also further supported by the finding that NAC did not reduce mitochondrial ROS (Supp. Fig. 3). Importantly, restoring cytosolic GSH with NAC^38^ blocked 6 MP mediated death of Leydig cells. These findings indicate 6 MP produces cytosolic ROS (which can be suppressed by NAC), a crucial step in eliciting Leydig cell death (Fig. 3d).

Because caspase 3 was strongly activated in Leydig cells by 6 MP treatment *in vivo* (see Fig. 2c), we investigated which caspase pathway was activated by 6 MP. Caspase 8 is typically part of the extrinsic apoptotic pathway, but caspase 8 inhibition (by Z-IETD-FMK) did not restore viability to 6 MP treated cells. In contrast, either caspase 9 inhibition (by Ac-LEHD-CHO, abbreviated LEHD) or pan-caspase inhibition (by ZVAD-FMK, abbreviated ZVAD) rendered Leydig cells refractory to the cytotoxic effects of 6 MP (Fig. 3e). To confirm that caspase 3 is activated in Leydig cells, we investigated whether a caspase 3 target, PARP, was cleaved. Using an antibody that specifically detects cleaved PARP, immunoblot analysis showed increased cleavage in 6 MP treated cells, consistent with caspase 3 activation. Further, inhibition of the upstream activator of caspase 3, caspase 9, suppressed 6 MP induced PARP cleavage (Fig. 3f). Intriguingly, 6 MP-induced ROS production was not attenuated by caspase inhibition (Fig. 3g). In total, these findings indicate ROS is produced by 6 MP, but is incapable of eliciting death alone and requires caspase 3 activation too.

We propose the following model for Leydig cell death by 6 MP generated ROS (Fig. 3h): 6 MP is metabolized to thiouric acid by xanthine oxidase. This, in turn, generates cytosolic ROS (suppressible by NAC) which, by acting upon mitochondria, produces cytochrome *c* release in a BAK-independent manner. Cytochrome *c* then binds to dATP bound Apaf1, inducing formation of an active oligomerized Apaf1/cytochrome *c* complex^39^. This complex, known as the apoptosome, recruits pro-caspase 9 which is then auto activated^40^. Activated caspase 9 then cleaves pro-caspase 3, initiating a cascade of events, like PARP cleavage, to produce cell death.

### Thiopurine nucleotides enhance apoptosome activation

**Figure 4:**
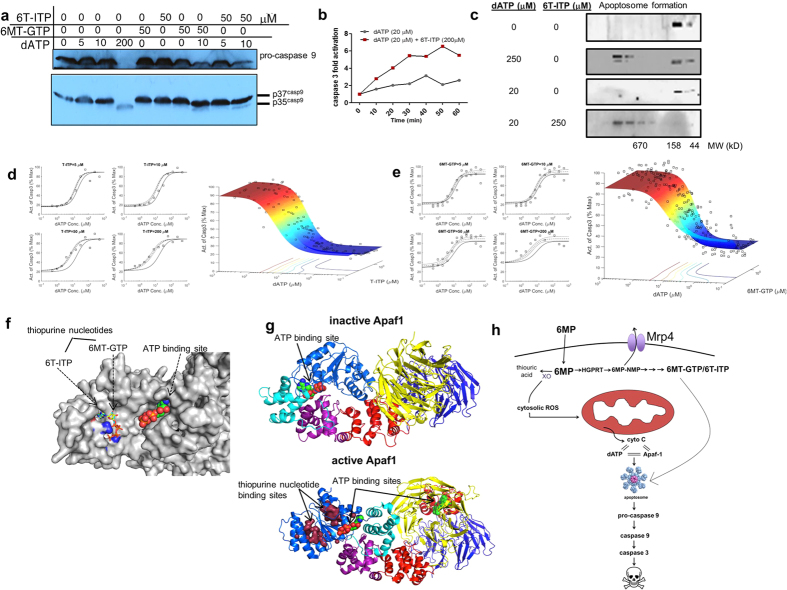
Thiopurine nucleotide triphosphates of 6 MP enhance Leydig cell death (**a**) Immunoblot of pro- and cleaved caspase 9 amounts in cytosolic extracts treated with varying concentration with 6T-ITP, 6MT-GTP, and dATP. (**b**) Time-dependent caspase 3 activation in cytosolic extracts treated with either dATP alone or dATP and 6T-ITP as indicated. (**c**) Apoptosome formation in the presence of dATP and/or 6T-ITP as visualized by immunoblot of Apaf1 in size exclusion fractions. (**d**) Activation of caspase 3 by 6T-ITP (α = 1.84), p < 10^−11^) and **(e)** 6MT-GTP (α = 0.44, p < 0.05). The solid curve represents the additive response. The dashed curve is the cross-section of the response surface model fit to the data and the dotted curves are the 95% confidence interval of the fitted curve. The corresponding 3D modeling plot, show the additive surface, is seen to the right of each respective group. (**f**) Structural model showing two individual binding sites for 6T-ITP (cyan) and 6MT-GTP (yellow) as well as dATP. (**g**) Apaf1 represented in both its inactive (right) and active (left) conformations. Thiopurine nucleotide binding sites are formed only in active Apaf1. A cross section of the response surface modeling plots of dATP plus varying concentrations (**h**) Model schematic showing the thiopurine nucleotide metabolites of 6 MP, along with dATP, bind and synergize with Apaf1, activating caspase 3 and promoting Leydig cell death.

In cell-free systems, some purine nucleoside triphosphate analogs can (by binding to Apaf-1) substitute for dATP to activate the apoptosome^41^,^42^. It is unknown if thiopurine nucleotides directly activate the apoptosome. Thiopurine nucleotide triphosphate metabolites are readily formed from 6 MP with the major intracellular triphosphates being 6-thio-inosine triphosphate (6 T-ITP) and 6-methylthio-guanosine triphosphate (6 MT-GTP)^43^. We hypothesized that 6 T-ITP and 6 MT-GTP might also substitute for dATP in promoting apoptosome activation. To test this hypothesis, we used gel-filtration to prepare purified cell-free apoptosome-containing extracts that were inactive due to the lack of endogenous nucleotides and cytochrome *c*^41,42^. The apoptosome was activated by supplementing with cytochrome *c* and dATP. Unlike dATP, thiopurine nucleotide triphosphates (up to 1000 μM) alone were incapable of activating the apoptosome as measured by caspase 3 activity (Supp. Fig. 4). Further, immunoblot analysis demonstrated that, as expected, physiological concentrations (5–10 μM) of dATP did not produce pro-caspase 9 cleavage (Fig. 4a). However, increasing dATP concentrations activated the apoptosome as shown by pro-caspase 9 cleavage (Fig. 4a). Intriguingly, when thiopurine nucleotides 6 T-ITP or 6 MT-GTP were combined with a non-apoptosome activating concentration of dATP, the apoptosome was activated (Fig. 4a). The accelerated rate of dATP-dependent apoptosome activation by 6 T-ITP (Fig. 4b), suggested enhanced apoptosome assembly.

### Thiopurine nucleotides enhance apoptosome assembly

Under non-denaturing conditions, the assembly of the apoptosome can be identified by monitoring the shift in the molecular weight of Apaf-1 from approximately 130 kDa to >670 kDa^44^. 6 T-ITP acceleration in the rate of dATP-dependent apoptosome activity (Fig. 4b), led us to hypothesize that 6 T-ITP might enhance apoptosome assembly. To interrogate apoptosome assembly, size-exclusion HPLC chromatography was performed (see Materials and Methods) (Fig. 4c). As expected, in the absence of cytochrome *c* addition, there was no apoptosome assembly (not shown). Notably, as a control, we showed that the overall profile of the proteins (represented by the UV trace of absorbance at 280 nm), eluted from the size-exclusion column, displayed no difference between inactive and active apoptosomes (Supp. Fig. 5). Immunoblot analysis of the size fractionated cytosol revealed Apaf1 in an apoptosome complex of >670 kDa^39^ in cellular extracts treated with 250 μM, but not 20 μM dATP (Fig. 4c). Strikingly, the addition of 6 T-ITP provoked apoptosome assembly by a non-apoptosome activating concentration of dATP (20 μM) (Fig. 4c).

Allosteric binding sites have the potential to affect catalytic activity, either synergistically or additively. To determine if thiopurine nucleotides activated the apoptosome, either synergistically or additively, extracts were treated with various combinations and concentrations of either 6 T-ITP and dATP or 6 MT-GTP and dATP (Fig. 4d,e). In general, increasing the concentration of either 6 T-ITP or 6 MT-GTP reduced the amount of dATP required for apoptosome activation (i.e., produced a left shift in the nucleotide dose-response curve). These dose-response data were then analyzed by “response surface” modeling^45^,^46^,^47^ to test for synergism or additivity. This analysis revealed that both thiopurine nucleotides tested, 6 T-ITP and 6 MT-GTP synergistically enhanced dATP (α = 1.84 p < 10^−11^; α = 0.44 p < 0.05, respectively) dependent apoptosome activation and revealed 6 T-ITP as a more potent activator (Fig. 4d,e).

As Apaf-1 has a single consensus nucleotide binding site, (G-(X)_4_-GKS, located between amino acids 154–161) that favors dATP over ATP^48^, we hypothesized that the synergistic apoptosome assembly provoked by thiopurine nucleotide triphosphates might be due to allosteric binding sites in Apaf-1. Using a “pocket searching” algorithm (see Methods), we developed an Apaf-1 structural model to investigate the possibility that thiopurine nucleotide triphosphates interacted with Apaf-1 at non-consensus binding sites (Fig. 4f). The pocket searching algorithm identified two potential thiopurine nucleotide binding sites. It is notable that these were only revealed when dATP was bound to Apaf-1. This model predicts that Apaf-1 thiopurine nucleotide binding sites are located at amino acid positions R205 and K198 for 6 T-ITP and 6 MT-GTP, respectively (Fig. 4g). However, these are only exposed after the transition to an dATP bound “active” conformation (Supp. Fig. 6). Notably, in this active conformation of Apaf-1, the predicted binding affinities for 6 T-ITP and 6 MT-6TP, at their cognate sites, were −7.51 kcal/mol and −7.73 kcal/mol, respectively; higher than their affinity for dATP (−6.21 kcal/mol).

Based on these findings, we propose a model of 6 MP induced Leydig cell death (Fig. 4h) that requires two concerted pathways. The initial metabolism of 6 MP to thiouric acid generates cytosolic ROS, producing cytochrome *c* release. In parallel, 6 MP is metabolized to thiopurine nucleotides which, by themselves, are incapable of activating the apoptosome. However, when Apaf-1 is loaded with dATP, a conformation is adopted rendering it capable of binding thiopurine nucleotides thereby promoting apoptosome activation. This new apoptosome activation mechanism underscores the importance of the relationship between thiopurine nucleotide concentrations and dATP in this novel mechanism of initiating cell death. Finally, thiopurine nucleotide activation of the apoptosome can be circumvented by MRP4 mediated export.

These studies are the first to show that 6 MP elicits Leydig cell death by a mechanism that requires the concerted action of xanthine oxidase-generated ROS and thiopurine nucleotide triphosphates. Undoubtedly, factors affecting either the amount of 6 MP generated ROS (related to the amount of xanthine oxidase^49^) or the amount of thiopurine nucleotides (e.g., affected by the amount of functional MRP4) (Supplemental Table 2) can dramatically impact Leydig cell survival. We suggest that the 6 MP-mediated mechanism described herein has the potential to contribute to the demise of proliferating cells too (e.g., cancer cells and hematopoietic cells). However, this unique mechanism was likely overlooked in previous investigations of 6 MP cytotoxicity, a consequence that might be related to prior studies emphasis on elucidating the DNA pathway of 6 MP mediated cell death^13^,^14^,^15^,^16^,^17^,^18^. We propose that this overlooked “apoptosome activation” pathway of thiopurine nucleotide induced death is likely to occur in proliferating cells too. One future challenge will be to dissect the relative contributions of the DNA and apoptosome pathway to 6 MP initiated cell death. Nonetheless, it is likely this pathway is extremely important in the 6 MP induced death and cytotoxicity of cells in non-proliferating tissues that are sensitive to 6 MP (e.g. liver and CNS). Finally, these findings have important implications not just for leukemia therapy, but also for patients that receive the 6 MP prodrug, azathioprine, for immunosuppression.

We propose that MRP4 function is critical in protecting Leydig cells against 6 MP induced cell death due to its inability to export 6 MP derived nucleotides. It is likely that individuals harboring functionally impaired variant alleles of MRP4 (Supplemental Table 2) might be at greater risk for 6 MP-induced LCF, and possibly, infertility. As some of these variants are frequently detected in certain populations, the incidence of 6 MP-induced LCF may be much higher in these vulnerable populations.

Another factor we had not originally considered was that the accumulation of 6 MP in Leydig cells might also be impacted by transporters mediating 6 MP uptake. The uptake of 6 MP can be mediated by several uptake carriers such as SLC28A2, SLC28A3, SLC29A1 and SLC29A2. We evaluated mRNA expression of these transporters in Leydig cells. Interestingly, among these four uptake carriers, Slc29a1 expression was the greatest in Leydig cells (Supp. Fig. 7). We speculate that increased Leydig cell uptake of 6 MP poses the same potential risk for LCF as deficiencies in the thiopurine nucleotide exporter, MRP4.

The reduced systemic testosterone and raised concentration of luteininzing hormone that characterizes LCF has been previously reported for childhood cancer survivors that received either high dose alkylating agent therapy or excess doses of radiotherapy^1^,^2^,^3^,^4^,^5^,^6^,^7^. To our knowledge, despite widespread chemotherapeutic use of 6 MP (and its pro-drug azathioprine), its contribution to LCF was previously unknown. However, clues in the literature suggested reproductive problems among those that received thiopurine based therapy^50^,^51^. Our studies revealed, for the first time, that some individuals that received 6 MP chemotherapy experienced profound LCF. We then showed, in an *in vivo* animal model, that this is due to 6 MP-induced cell death of Leydig cells. Because adult Leydig cells do not replicate their DNA (and thiopurine incorporation into DNA is the typical mechanism of cell death^13^,^14^,^15^), mechanistic studies were conducted to investigate how Leydig cells were killed by thiopurines. We demonstrated that thiopurines mediate cell death by a unique mechanism that activates the apoptosome. The activation of the apoptosome by 6 MP in Leydig cells required both formation of thiopurine nucleotides and ROS. This new mechanism provides insights into approaches (such as reducing ROS by blocking 6 MP metabolism by xanthine oxidase) that might be therapeutically exploited to mitigate 6 MP damage to Leydig cells and reduce the risk of thiopurine-induced LCF and infertility.

## Methods

### Patients

After review and approval by the St. Jude Children’s Research Hospital Institutional Review Board, all participants were informed, consented and enrolled in the SJLIFE protocol^4^ and all methods were carried out in accordance with the approved guidelines. Inclusion criteria included treatment for a childhood malignancy at St. Jude Children’s Research Hospital, age ≥18 years and >10 years post-diagnosis of cancer. Clinical and laboratory evaluations were performed between 2007 and 2012. Patient and treatment data were extracted from the medical records. The records of patients with a diagnosis of Leydig cell failure (LCF) were subsequently reviewed in order to identify those whose treatment exposures included 6-MP and MTX and who were not exposed to alkylating agent chemotherapy and/or testicular radiotherapy. The cumulative doses of MTX and 6-MP are shown in Supplementary Table 1.

The diagnosis of LCF was based either on a known history of having this condition or because laboratory assessments showed plasma total testosterone levels <250 ng/dL concurrent with luteinizing hormone (LH) levels >7 IU/L at two separate occasions. Total testosterone and LH levels were measured using electro-chemiluminescent immunometric assays (Roche Cobas 6000 analyzer, Roche Diagnostics, Indianapolis, IN, USA) at a minimum of two independent times.

### Animals

The Mrp4 mice used in this study were generated in our laboratory as previously described^52^ on a mixed C57BL6/129-SVJ background and were maintained by intercrossing littermates.

### *In vivo* 6 MP injections

Age-matched WT and KO mice received daily IP injections of 50 mg/kg 6 MP for 10 days with saline treated control mice treated likewise. All mice were euthanized 24 hours following the final injection and testes were harvested for analysis.

### Immunohistochemistry

At the St. Jude Veterinary Pathology Core, formalin-fixed, paraffin-embedded slides of testes harvested following 6 MP injection were prepared by standard methods and stained with antibodies to hematoxylin and caspase 3 using the LabVision720 autostainer (ThermoShandon). For both caspase 3 positive cells and interstitial space determinations, entire slides were counted at ×20 magnification.

### Leydig cell culture

Leydig cells were isolated from adult Mrp4 mice as previously described^53^. Cells were cultured in DMEM/F12 (1:1) media (Life Technologies) supplemented with 5 μg/ml bovine insulin (Sigma), 2.5 μg/ml transferrin (Sigma), and 10 μg/ml pen-strep (Life Technologies). Cultured Leydig cells were incubated at 37 °C for varying intervals and treatment conditions as indicated in the figure legends.

### Leydig cell viability

For viability assays, Leydig cells were cultured at a density of 10^6^ cells per ml in a six-well plate and incubated with indicated drugs for 12 hours. Subsequently, the cells were harvested from the plate with a cell lifter and viability immediately assessed Trypan Blue dye exclusion. All experiments were performed in triplicate.

### ATP measurement

ATP concentrations were assessed using the ATP Bioluminescent Assay Kit (Sigma-Aldrich). Briefly, Leydig cells were isolated from Mrp4 animals and cultured overnight at 37° at a density of 5 × 10^5^ cells per well in a 12-well plate. Cells were treated with 500 μM 6 MP for one hour, washed with 1× phosphate buffered saline (PBS), then incubated with ATP lysis buffer (100 mM potassium phosphate buffer (pH 7.8), 1% Triton X-100, 2 mM EDTA, and 1 mM dithiothreitol) for 30 minutes at 37°. 2,4-Dinitrophenol (DNP) was used as a positive control^54^. Luminescence was measured using the Clarity Luminescence Microplate Reader (BioTek Instruments).

### Mitochondrial membrane potential

Leydig cells were isolated from Mrp4 mice, seeded at a density of 5 × 10^5^ cells per well in a 96-well plate, and cultured overnight. Cells were treated with 500 μM 6 MP for two hours or 200 μM DNP. Fifteen minutes prior to the end of the incubation time, 25 nM tetramethylrhodamine ethyl ester (TMRE) (Life Technologies) was added. TMRE florescence was immediately evaluated using a Hybrid H4 Multi-Mode microplate reader (BioTek Instruments) at an excitation of 485 nm and emission of 595 nm (Ex485/Em595).

### Mitochondrial isolation

Isolated Leydig cells were cultured for 12 hours in either 500 μM 6 MP or vehicle (PBS). Cells were then scraped, pelleted at 1800 × g for 15 min at 4 °C, and washed with 1× PBS and spun again. The cells were then re-suspended in ice-cold hypotonic RSB (10 mM NaCl, 1.5 mM MgCl_2_, 10 mM Tris buffer (pH 7.5), and 1 tablet Roche protease inhibitor per 50 ml) and transferred to a glass Dounce homogenizer. Following a 10 min incubation on ice, a size B, loose pestle was used to disrupt cellular structure. The homogenization was spun at 1300 × g for 5 min at 4 °C, supernatant collected and re-precipitated by centrifugation at 10000 × g for 15 min at 4 °C. The resulting pellet was washed in 1× MSB (210 mM mannitol, 70 mM sucrose, 5 mM Tris buffer (pH 7.5), and 1 mM EDTA) and centrifuged at 10000 × g for 15 min at 4 °C. The resulting mitochondrial pellet was then re-suspended in 1× MSB.

### Cytochrome *c* release assay

Isolated Leydig cells were plated at a density of 10^7^ cells in a p100 dish, with a total of 10^8^ cells used for each treatment. Cells were either left untreated or incubated with 500 μM 6 MP for 12 hours and were then harvested and mitochondria isolated per the previously described protocol. To obtain the cytosolic fraction containing the released cytochrome *c*, the two final supernatants were pooled together. Protein concentration was determined by the Bradford assay method, and samples were size fractionated by SDS-PAGE. Both untreated and 6 MP-treated Leydig cells were assayed for cytochrome *c* (Santa Cruz Biotechnology) and Vdac (Abcam) was used to confirm the mitochondrial fraction. Bands were quantitated against an endogenous control band.

### Immunoblotting

Isolated WT Leydig cells were cultured in 100 mM dishes and either untreated or treated with 500 μM 6 MP for 12 hours. Cells were harvested with a cell lifter and centrifuged at 1800 × g for 5 minutes at 4 °C, washed with 1× PBS, and re-precipitated by centrifugation. The Leydig cells were ultrasonically disrupted in M-Per (Thermo Scientific) plus a protease inhibitor mixture (Roche Diagnostics). Protein concentrations were determined by the Bradford assay method, and extracts were size fractionated by SDS-PAGE. Untreated and 6 MP treated Leydig cells were assayed for p53, PARP, Bcl-XL (Cell Signaling Technology), Bak (Upstate Biotechnology), Bcl-2 (BD Pharmingen), Mcl1 (Rockland Immunochemicals), and cytochrome *c* (Santa Cruz Biotechnology). Relative protein quantities were determined by densitometry using ImageJ software.

### Reactive oxygen species (ROS) measurement

Leydig cells were isolated from WT mice, seeded at a density of 2.5 × 10^4^ cells/well in a 96 well plate, and cultured overnight. The following day, cells were pre-treated for 30 minutes with 2′,7′-dichlorofluroescein diacetate (DCFDA) and then exposed to 6 MP for 3 hours at 37 °C. Hydrogen peroxide (1 mM) was utilized as a positive control for ROS production. Following the 3-hour incubation, ROS was measured using the BioTek Synergy HT Multi-Mode Microplate Reader (BioTek Instruments) at Ex485 nm/Em535 nm. Mitochondrial ROS was measured with the MitoSOX reagent according to the manufacturer’s instructions.

### Caspase 3 activation

Purified cytosolic extracts were prepared from 2–5 × 10^8^ MEL cells. MEL cells were collected, centrifuged for 5 minutes at 1500 × g and washed with 1× HBSS. After precipitation, the cells were then re-suspended in 1–2 ml activation buffer (50 mM PIPES (pH 7.0), 20 mM KCl, 2 mM MgCl_2_, 5 mM EDTA, 1 mM DTT, and 1× protease inhibitor (Roche)). The cell suspension was frozen on dry ice, thawed, and then subjected to 70 strokes with a loose glass homogenizer before being centrifuged twice for 10 minutes at 14000 × g. The final supernatant was passed through a 30 kD Amicon Ultra filtration unit (Millipore) at 5600 × g with buffer replacement to remove endogenous nucleotides. Optimal amounts of protein for use in the assay were determined empirically with each extract but were frequently in the range of 100–200 μg per reaction. Extracts were incubated in 96 well plates with the desired combination of nucleotides and cytochrome *c* in a volume of 50 μM at 37 °C and were then brought to 100 μl volume with 2× caspase 3 substrate mix (50 mM PIPES (pH 7.0), 20% glycerol, 10 μM DEVD-AMC, 1 mM DTT, 0.5 mM EDTA, 0.02% NP40, and 1× protease inhibitor). Caspase 3 activity was measured by monitoring fluorescence (Ex380/Em460). Negative controls were incubated only with cytochrome *c* (4 μM).

### Apaf-1 modeling

In order to model the potential nucleotide binding sites of Apaf-1, the nucleotide binding domains of Apaf-1 in both active (bound with ATP, PBD ID: 3J2T) and inactive (bound with ADP, PBD ID 3SFZ) states were isolated from two deposited structures and optimized by Sybyl (Tripos Inc). A pocket searching algorithm using ICM software^55^ was used. Once the pocket was identified, all selected compounds were docked into the site binding. Affinities were evaluated using the Glide module (Schrodinger Inc.)^56^,^57^ with standard precision.

### Response surface modeling

Matlab, version R2014a, Mathworks, was used to quantify the combined effects of dATP and 6 T-ITP or 6 MT-GTP on apoptosome activation. A combination was considered either synergistic or antagonistic if the interaction term (α) describing the change in response relative to the additive model was either positive or negative, respectively. The combination was considered different from additive if both the interaction coefficients were significantly different than zero.

### Study Approval

All animals were housed and fed under identical conditions. All experiments in this study were approved by the St. Jude Animal Care and Use Committee and all methods were carried out in accordance with the approved guidelines.

## Additional Information

**How to cite this article**: Morgan, J. A. *et al.* Apoptosome activation, an important molecular instigator in 6-mercaptopurine induced Leydig cell death. *Sci. Rep.* **5**, 16488; doi: 10.1038/srep16488 (2015).

## Supplementary Material

Supplementary Information
