# Supplementary material for: Apoptosome activation, an important molecular instigator in 6-mercaptopurine induced Leydig cell death
Source: Sci Rep. 2015 Nov 18;5:16488. doi: 10.1038/srep16488 (PMC4649703; doi:10.1038/srep16488)
Supplement: Supplementary Information [file srep16488-s1.pdf]

Apoptosome activation, an important molecular instigator in 6-mercaptopurine induced Leydig cell death

Jessica A. Morgan[1], John Lynch[1], John C. Panetta[1], Yao Wang[1], Sharon Frase[2] Ju Bao[3], Jie Zhang[3], Joseph T. Opferman[4], Laura Janke[5], Daniel M. Green[6], Wassim Chemaitilly[6,7], and John D. Schuetz[1]

Departments of [1]Pharmaceutical Sciences, [2]Cellular Imaging Shared Resource, [3]Structural Biology, [4]Cell and Molecular Biology, [5]Pathology, [6]Epidemiology & Cancer Control, and [7]Endocrinology, St. Jude Children's Research Hospital, 262 Danny Thomas Place, Memphis, TN 38105.

Supplemental Figure 1

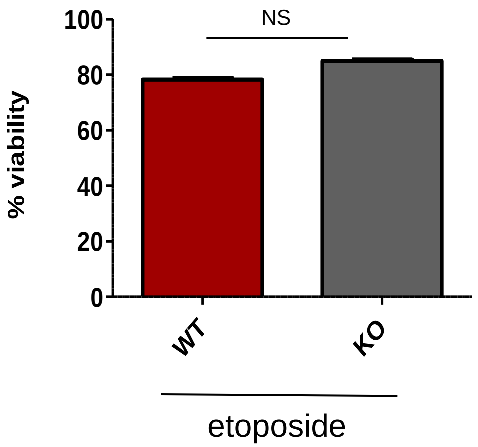

Supplemental Figure 2

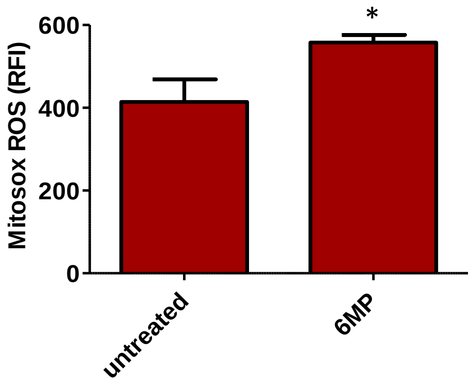

Supplemental Figure 3

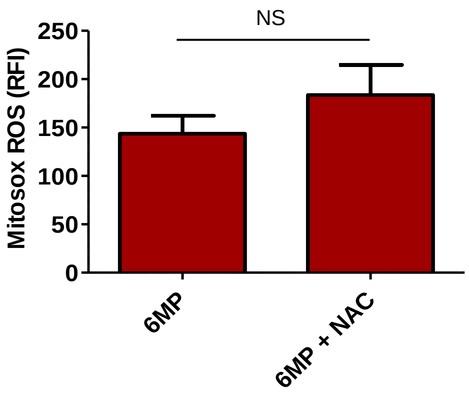

Supplemental Figure 4

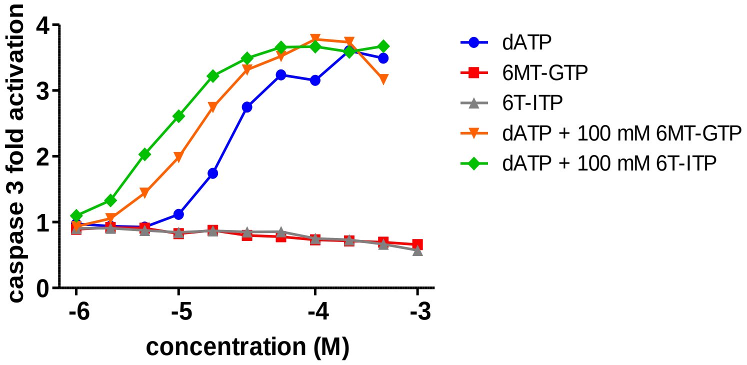

Supplemental Figure 5

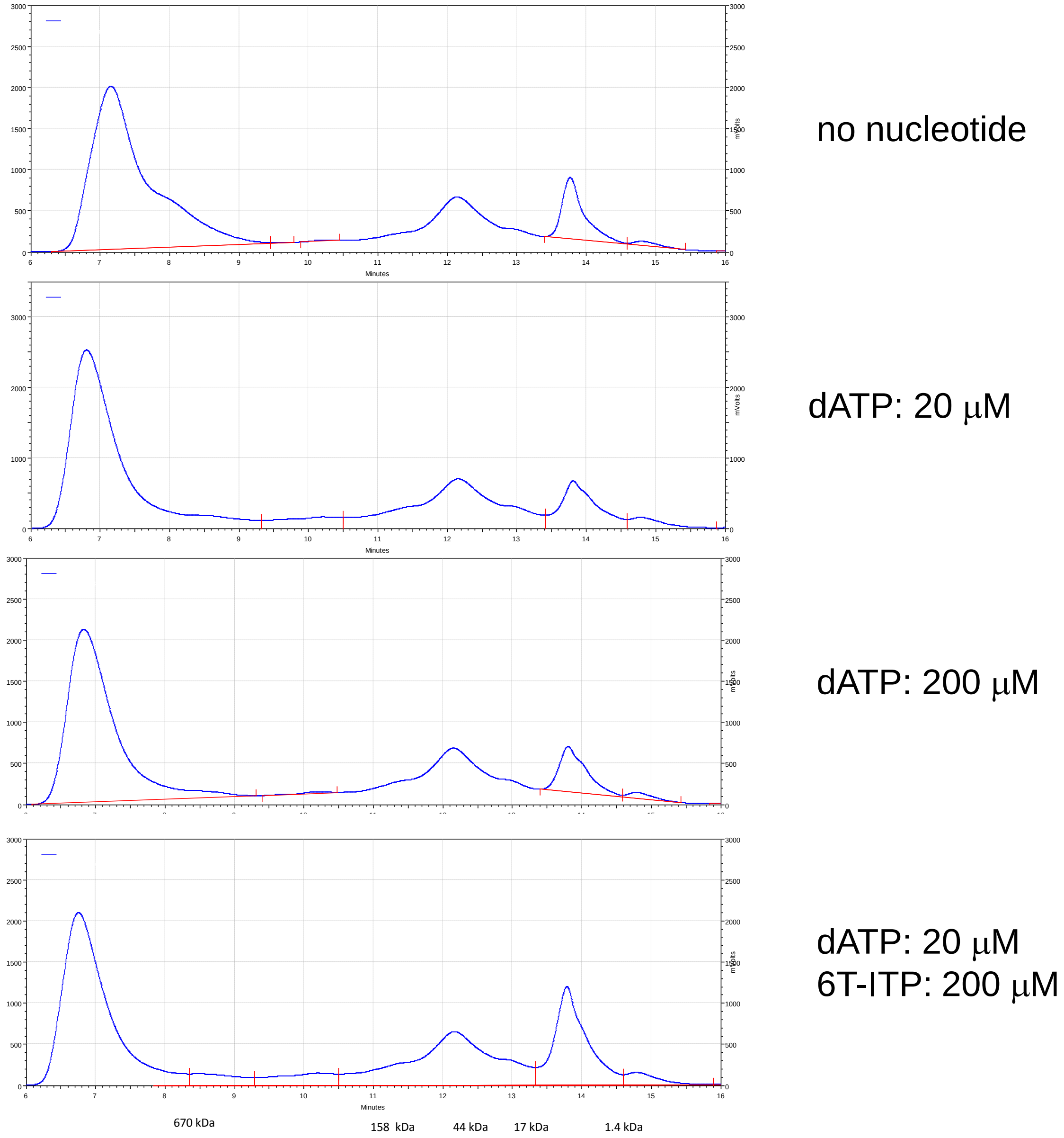

Supplemental Figure 6

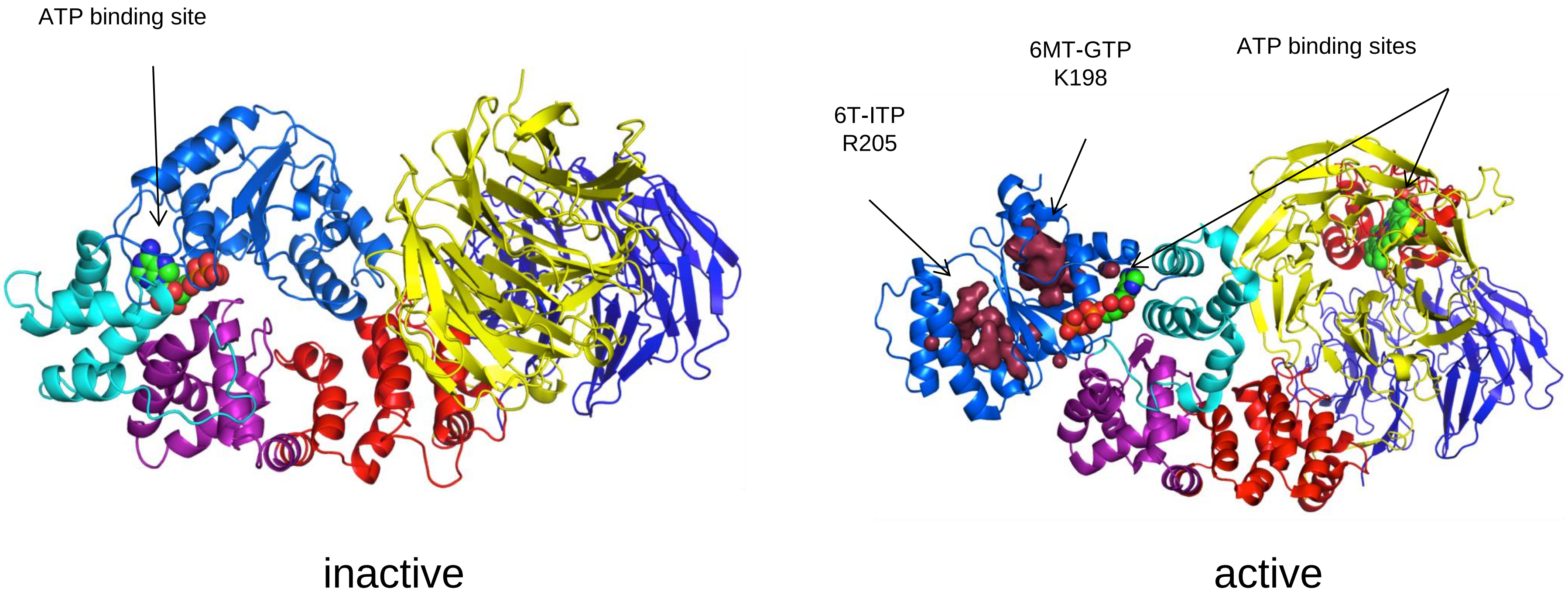

Supplemental Figure 7

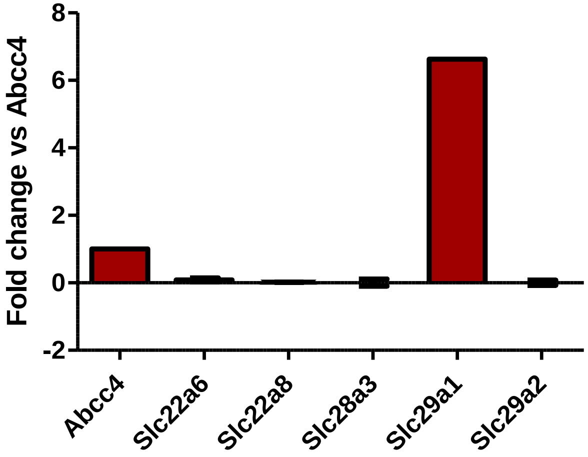

Supplemental Table 1

	Patient1	Patient2	Patient3	Patient4
Oral 6MP, dose received*	40999	48038	38348	46781
Oral 6MP, maximum protocol dose*	45600	45600	42000	42000
Oral MTX, dose received*	3948	0	3949	5167
Oral MTX, maximum protocol dose	0	0	1600	1600
IV MTX, dose received*	386	2762	0	0
IV MTX, maximum protocol dose*	2600	2600	0	0
IT MTX, dose received*	50	60	249	203
IT MTX, maximum protocol dose*	180	180	192	192
Plasma LH, IU/L, first evaluation	8.4	8.7	8.4	7.9
Plasma total testosterone, ng/dL, first evaluation	164	69	193	173
Plasma LH, IU/L, second evaluation	7.6	9.9	n/a	7.0
Plasma total testosterone, ng/dL, second evaluation	160	145	170	154

*=mg/m2 of body surface area; LH= luteinizing hormone, Yellow highlight indicates protocol dosage. All doses are cumulative.

Supplemental Table 2

Variant	Nucleotide Position	Allele Frequency		
		**EA**	**AA**	**ALL**
G2269A	E757K	1.01	0.32	0.8
G559T	G187W	2.28	0.66	1.7
G912T	K304N	7.04	17.1	10.5
C232G	P78A	0.0116	0	0.0077
C3425T	T1142M	0.83	0.068	0.569
G1460A	G487E	NA	NA	NA
G2596A	I866V	.674	.1	.476
G2560T	V854F	.569	.204	.44
C52A	L18I	2.05	1.27	1.79
EA: European American, AA: African American, NA: not available				

Supplemental Figures

Supplemental Figure 1: Etoposide does not produce Leydig cell death Viability of WT Leydig cells treated with either 500 μM 6MP or 100 μM etoposide for 12 hours. Experiment performed in duplicate. NS = not significant.

Supplemental Figure 2: 6MP slightly increases mitochondrial ROS Measurement of mitochondrial ROS using fluorescent substrate MitoSOX in untreated and 6MP treated Leydig cells. Experiments performed in duplicate. All error bars are mean ± SEM. $^*p \leq 0.05$.

Supplemental Figure 3: NAC does not inhibit mitochondrial ROS formation Mitochondrial ROS as measured by the florescent substrate MitoSOX in 6MP treated or 6MP plus NAC treated cells. H_2O_2 was used as a positive control for mitochondrial ROS generation. Experiments performed in duplicate.

Supplemental Figure 4: Thiopurine nucleotides alone do not activate caspase 3. Caspase 3 activation in MEL extracts treated with varying concentrations of dATP, 6MT-GTP, and 6T-ITP alone or in combination at the indicated concentrations. Graph shows a representative experiment performed three times with similar results.

Supplemental Figure 5: Active and inactive apoptosome complexes give similar HPLC size exclusion protein elution profiles. A280 tracing generated using size

exclusion high performance liquid chromatography examining inactive apoptosome complexes (dATP only) and active complexes (dATP and 6T-ITP).

Supplemental Figure 6: Apaf-1 modeling shows distinct binding site for two nucleotide metabolites Inactive Apaf-1 (left) contains a single dATP binding site with no other nucleotide binding sites displayed. Active Apaf-1 (right), bound with cytochrome *c* (not shown) and dATP reveals a second potential dATP binding site as well as sites available for 6T-ITP (R205) and 6MT-GTP (K198). A second alpha helical domain (shown to the right of the model in red) appears in active Apaf-1 as it is not present in the inactive form. The model was optimized using Sybyl and nucleotide binding affinities were evaluated using the Glide module (see Methods).

Supplemental Figure 7: Mrp4 uptake transporter, Slc29a1, is increased in Leydig cells RNA expression of various Mrp4 uptake carriers in pooled Leydig cell samples as determined by microarray analysis.
